# Association of dietary inflammatory index on all-cause and cardiovascular mortality in U.S. adults with metabolic dysfunction associated steatotic liver disease

**DOI:** 10.3389/fnut.2025.1478165

**Published:** 2025-04-01

**Authors:** Lin Tao, Tiantian Wu, Xiaoning Du, Qian Li, Yuefei Hao, Tao Zhou, Yinping Yi

**Affiliations:** Henan Provincial People’s Hospital, Zhengzhou, China

**Keywords:** MASLD, dietary inflammatory index, cardiovascular, mortality, NHANES

## Abstract

**Backgrounds:**

An inflammatory diet is pivotal in metabolic dysfunction-associated steatotic liver disease (MASLD) development. However, it remains unclear whether Dietary Inflammatory Index (DII), which serves as a reliable indicator to assess pro-inflammatory diet, have associative effects on mortality outcomes of MASLD.

**Methods:**

Participants in the National Health and Nutrition Examination Survey (NHANES) database from 1999 to 2018 years were included. Kaplan–Meier (KM) curves were used to estimate survival probabilities, while Cox regression analysis and restricted cubic splines (RCS) were employed to assess the association between DII and mortality outcomes. The concordance index (C-index) evaluated the accuracy of multivariate-adjusted DII for mortality among MASLD participants.

**Results:**

The cohort consisted of 4,510 men and 4,323 women with a median age of 52 years. Multivariate-adjusted Cox regression analysis revealed that high levels of DII were significantly associated with the all-cause mortality of participants with MASLD (multivariable-adjusted hazard ratio (aHR) = 1.28, 95% confidence interval (CI) 1.10–1.49, *p* = 0.002, DII aHR for cardiovascular mortality = 1.28, 95% CI 1.07–1.53, *p* = 0.006). The C-index for the multivariate model, integrating DII and other clinical variables, was 0.837 for all-cause mortality and 0.860 for cardiovascular mortality. RCS analysis showed a positive linear relationship between DII and all-cause mortality rate (*p* for nonlinearity = 0.057), with no significant nonlinearity for cardiovascular mortality (*p* = 0.953). Subgroup analyses indicated stronger associations in participants <65 years, married, with a college education, non-smokers, non-drinkers, and those without hypertension.

**Conclusion:**

Elevated DII levels are linked to higher mortality in adults with MASLD, underscoring the index’s utility in predicting mortality risks. These findings shows that dietary interventions targeted inflammation may be helpful in this population.

## 1 Introduction

Metabolic dysfunction-associated steatotic liver disease (MASLD), previously termed non-alcoholic fatty liver disease (NAFLD), was officially renamed in June 2023 (1). The prevalence of MASLD is rising rapidly, affecting 32.4% of the population in 2022, making it one of the most common chronic liver diseases (2, 3). MASLD is characterized by the accumulation of fat in hepatocytes, excluding the impact of viruses, alcohol, and autoimmune factors (4). It affects over one-third of the global population and is associated with significant morbidity and mortality due to its progression to metabolic dysfunction-associated steatohepatitis (MASH), cirrhosis, and hepatocellular carcinoma. Beyond liver-specific outcomes, MASLD contributes to systemic metabolic dysfunction, increasing the risk of cardiovascular disease and all-cause mortality (5).

Cardiovascular complications and systemic metabolic dysfunction are the primary causes of mortality in individuals with MASLD (6). Given the strong links between diet, inflammation, and metabolic dysfunction—key drivers of MASLD progression—dietary management is a cornerstone of nonpharmacological strategies for MASLD. Dietary patterns with low inflammatory potential may help mitigate disease progression and improve health outcomes. Additionally, bioactive compounds, such as polyphenols and omega-3 fatty acids, have demonstrated anti-inflammatory properties that may support liver health in MASLD populations (7, 8). However, despite the potential benefits of specific dietary components, the overall inflammatory potential of the diet and its impact on MASLD-related health outcomes remain insufficiently explored.

To address this, the Dietary Inflammatory Index (DII) has been widely applied as a tool to quantify dietary inflammatory potential, providing a standardized assessment of the inflammatory impact of an individual’s diet. Developed by Shivappa et al. in 2014 (9), the DII quantifies the effects of dietary components on inflammatory markers such as IL-1β, IL-6, TNF-*α*, and C-reactive protein, assigning each food parameter a score based on its pro-inflammatory or anti-inflammatory properties. Higher DII scores denote a diet with greater inflammatory potential, whereas lower scores reflect anti-inflammatory effects.

Studies in populations with obesity and aging have linked higher DII scores to increased mortality, highlighting the impact of dietary inflammation in metabolically compromised populations. For example, in a prospective cohort study involving 3,521 adults within the normal-weight BMI range, a pro-inflammatory diet, as indicated by high DII scores, was associated with an increased risk of cardiovascular disease (CVD) mortality among those with central obesity (10). Another study utilizing data from the National Health and Nutrition Examination Survey (NHANES) found that lower DII scores were linked to a decreased risk of all-cause mortality in aging populations (11). Moreover, MASLD is a systemic metabolic disorder that significantly increases the risk of cardiovascular disease-related mortality and various malignancies (12, 13). Unlike general obesity or aging populations, individuals with MASLD exhibit distinct inflammatory profiles driven by hepatic steatosis, insulin resistance, and metabolic dysregulation. Given the chronic inflammatory state of MASLD, the DII not only assesses dietary inflammation but also reflects how pro-inflammatory diets may exacerbate existing inflammation and accelerate disease progression. A high DII diet can increase pro-inflammatory cytokines (IL-6, TNF-*α*), oxidative stress, and gut dysbiosis, further aggravating hepatic inflammation and metabolic dysfunction. Thus, DII serves as a key marker for both dietary inflammation and its role in MASLD progression.

Given the significant burden of MASLD and its progression to more severe liver diseases and associated comorbidities, it is critical to investigate how dietary inflammatory potential, as measured by DII, impacts all-cause and cardiovascular mortality in this population. The present study employed data from the NHANES from 1998 to 2018. The primary objective was to examine the association between DII and all-cause and CVD mortality in United States adults diagnosed with MASLD. By analyzing this relationship, we hope to provide insights that could inform dietary recommendations and interventions aimed at reducing inflammation and improving long-term health outcomes in this population.

## 2 Materials and methods

### 2.1 Data source

Data for this study were derived from the NHANES database between 1999 and 2018. NHANES is a program of studies designed to assess the health and nutritional status of adults and children in the United States, conducted by the National Center for Health Statistics (NCHS) as part of the Centers for Disease Control and Prevention (CDC). NHANES employs a stratified, multistage probability sampling design to produce a sample that is representative of the noninstitutionalized U.S. population (14). To ensure high data quality, NHANES uses rigorous standardized protocols in data collection, including thorough training for interviewers and examiners, regular calibration of measurement equipment, and standardized data collection environments. Extensive quality control procedures, such as double-checking entries and cross-referencing data, are applied throughout the process to maintain accuracy and validity. Furthermore, NHANES data is continually reviewed and updated to reflect changes in population demographics and health trends, making it a widely trusted resource for epidemiological research. Specific description of the NHANES database can be found on the website.^1^

### 2.2 Participants selection

**Figure 1:**
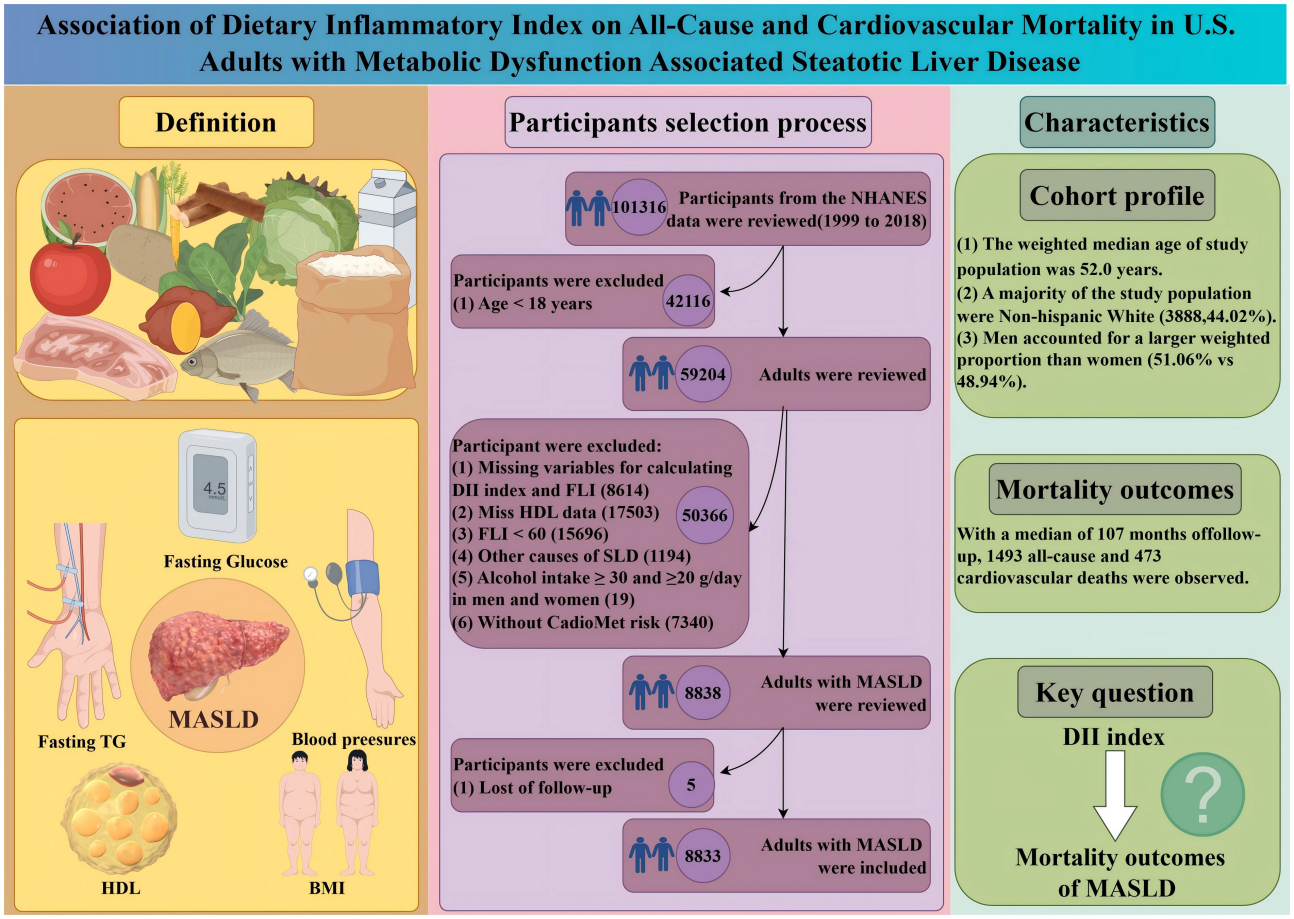
Scheme of the aim of the study and participants selection process. We aim to evaluate the association between varied DII with the mortality outcomes of adults with MASLD. MASLD, metabolic dysfunction-associated steatotic liver disease; DII, diet infammatory index; BMI, body mass index; HDL, high-density lipoprotein; TG, triglyceride. Other protential causes of SLD: viral hepatitis, autoimmune liver disease, genetic liver diseases, drug- or medication-induced liver disease, and alcohol-related liver disease.

To evaluate the association between the DII and all-cause and cardiovascular mortality of adults with MASLD, only participants with the diagnosis of MASLD were included. Therefore, during the 10 cycles of interviews (1999 to 2018), 101,316 participants were reviewed. After excluding the participants aged below 18 years old, there were 59,204 participants left. Additionally, participants with missing data on DII, body measurements, and metabolic dysfunction-associated factors were excluded. Then, participants presenting clinical features of FLI < 60, with other causes of SLD, a history of moderate to heavy alcohol intake, lacking cardiometabolic risk factors, or lost to follow-up were further excluded. Finally, there were 8,833 participants with MASLD were included in this study (Figure 1).

Participants with missing data for critical variables, such as FLI, DII, and key metabolic dysfunction-associated factors, were excluded. However, minor missingness in covariates used in the regression models was addressed using multiple imputation, as described in Section 2.5.

### 2.3 Assessment of MASLD

Direct ultrasonographic assessments of hepatic steatosis were missing in most of the interview cycles. Thus, hepatic steatosis was determined by using the fatty liver index (FLI), which was a reliable tool to evaluate steatotic liver disease (SLD), with high sensitivity and specificity (15, 16). The equation is listed below (17):


$$FLI=\left(e^{0.953\times \ln\left(TG\right)+0.139\times BMI+0.718\times \ln\left(GGT\right)+0.053\times WC-15.745}\right)/\left(1+e^{0.953\times \ln\left(TG\right)+0.139\times BMI+0.718\times \ln\left(GGT\right)+0.053\times WC-15.745}\right)\times 100$$

TG refers to triglyceride, GGT refers to gamma-glutamyl transferse, BMI refers to body mass index, and WC refers to waist circumference (15, 17). Based on prior research, participants with an FLI below 60 were deemed unlikely to have hepatic steatosis, whereas those with an FLI of 60 or higher were considered likely to have the condition. Therefore, participants with FLI ≥ 60 were diagnosed with SLD (18).

To meet the diagnostic criteria for MASLD, participants with viral hepatitis, autoimmune liver disease, genetic liver disorders, drug- or medication-induced liver disease, alcohol-related liver disease, as well as individuals whose daily alcohol intake met or exceeded 30 g/day for men and 20 g/day for women, were excluded. Consequently, MASLD was defined as SLD with a combination of the presence of at least one cardiometabolic risk factor (19):

(1) BMI ≥ 25 kg/m^2^ or WC ≥ 94 cm for males and ≥ 80 cm for females;
(2) FBG ≥ 100 mg/dL or 2-h post-load glucose levels ≥140 mg/dL or hemoglobin A1c ≥ 5.7% or diabetes mellitus (DM) or undergoing hypoglycemic therapy for DM;
(3) Blood pressure ≥ 130/85 mmHg or undergoing antihypertensive drug treatment;
(4) Fasting plasma triglycerides ≥150 mg/dL or undergoing lipid-lowering treatment;
(5) Plasma HDL-cholesterol <40 mg/dL for males and < 50 mg/dL for females or undergoing lipid-lowering treatment.

### 2.4 DII measurements

The DII is a scoring index developed to quantify the overall inflammatory potential of an individual’s diet. This index was created by Shivappa et al., who conducted an extensive review of the literature to determine the impact of various foods and dietary constituents on six inflammatory markers: IL-1β, IL-4, IL-6, IL-10, TNF-*α*, and CRP. The DII assigns pro-inflammatory and anti-inflammatory scores to each food parameter based on the findings of these studies. These scores are then weighted to obtain an overall inflammatory effect score for each specific food parameter.

A lower DII score indicates a diet with a greater capacity to reduce inflammation, whereas a higher DII score suggests a diet with a greater capacity to promote inflammation. In this study, the DII was computed using data from the NHANES database, specifically from 24-h dietary recall (24HDR) data, which were collected during the health examination phase.

A total of 27 nutrients were utilized in the computation of the DII score, including alcohol, vitamins B_12_/B_6_, *β*-carotene, caffeine, carbohydrates, cholesterol, total fat, fiber, folate, iron, magnesium, zinc, selenium, monounsaturated fat acid (MUFA), niacin, n-3 fatty acids, n-6 fatty acids, protein, polyunsaturated fatty acid (PUFA), riboflavin, saturated fat, thiamin, and vitamins A/C/D/E.

Although the DII can evaluate the inflammatory effects of up to 45 food components, prior studies have demonstrated that using fewer than 30 food components does not significantly affect the DII’s evaluative ability (20). In this study, the NHANES data allowed for the use of 27 food parameters to calculate the DII, ensuring a comprehensive assessment of the dietary inflammatory potential.

### 2.5 Clinical characteristics and covariates

We collected the demographic characteristics of participants with MASLD from the NHANES database.

Sociodemographic characteristics, including gender (male or female), age, race/ethnicity (Hispanic, non-Hispanic White, non-Hispanic Black, or other races), marital status (not married, married), education level (≤ high school, college, or > college), and poverty income ratio (PIR: < 1.3, 1.3–3.5, > 3.5), were obtained during the household interview phase. PIR was defined as the ratio of family income to the federal poverty threshold, adjusted for family size.

Physical and laboratory data collected during the health examination phase included measurements of waist circumference (WC), height, BMI, triglycerides (TG), total cholesterol (TC), glutamic-pyruvic transaminase (ALT), and high-density lipoprotein (HDL).

Hypertension status was defined using a combination of self-reported physician diagnoses (household interview phase), antihypertensive medication use, and blood pressure measurements taken during the health examination phase (systolic blood pressure ≥ 130 mmHg or diastolic blood pressure ≥ 85 mmHg).

### 2.6 Outcome measurements

The main outcome of this study was the all-cause mortality of participants with MASLD. The secondary outcome was the cardiovascular mortality of the participants with MASLD. The ICD-10 (International Statistical Classification of Diseases, 10^th^ revision) was used to check the causes of mortality. The mortality of the follow-up population were obtained from the NHANES public-use linked mortality file as of December 31, 2019, which was correlated with the National Death Index (NDI) through a probability matching algorithm. The period of follow-up was calculated from the date when the interview was initially taken to either the date of the patient’s death or December 31, 2019 (21).

### 2.7 Statistical analysis

According to the analytic guideline of the NHANES database^2^ (accessed on 17 June 2024), NHANES’ person-level sample weights were applied to adjust for the multistage sampling design, which includes stratification and clustering. These adjustments ensure that the results are representative of the U.S. adult population with MASLD and provide accurate variance estimates.

To address missingness in secondary covariates, multiple imputation by chained equations (MICE) was employed. Five imputed datasets were generated, incorporating all variables used in the regression models. Missingness for covariates, such as PIR, blood pressure, and laboratory measures (e.g., HDL and ALT), was typically below 10%. The pooled results from imputed datasets were combined using Rubin’s rules to ensure robust inference and minimize bias.

In this study, continuous variables were summarized as medians with interquartile ranges (IQRs) to represent their central tendency and variability. Categorical variables were presented as frequencies with weighted percentages. Comparisons of continuous variables between survivors and non-survivors were conducted using the Kruskal–Wallis test. Categorical variables were compared using the Chi-Squared test to assess proportional differences.

Cox proportional hazard models were used to estimate the association of DII with all-cause and cardiovascular mortality of the participants with MASLD. The selections of controlled covariates in the present study were based on previous literature evaluating the survival of MASLD (22–24). Specifically, model 1 served as the unadjusted analysis. Besides, brief adjustments for age, gender, and race were made in Model 2. In the fully adjusted model, we accounted for age, gender, race, marital status, educational level, poverty income ratio (PIR), plasma glucose concentration, alcohol use, BMI, serum levels of TC, ALT, HDL and TR. The concordance index (C-index) (25) was employed to assess the model accuracy of the multivariate-adjusted DII indices for mortality outcomes among participants with MASLD. Kaplan–Meier (KM) curves with NHANES sample weights were used to illustrate survival patterns across quartiles of the DII among participants with MASLD. For cardiovascular mortality, the cumulative incidence function (CIF) was used to account for competing risks, calculated with the cmprsk package in R.

To evaluate potential non-linear associations between the DII and mortality outcomes, restricted cubic splines (RCS) with three knots were employed. The selection of knots for the RCS curves was guided by the minimization of Akaike’s Information Criterion (AIC). A likelihood ratio test was used to compare the spline model to a linear model. *p*-values for non-linearity were reported, with insufficient evidence for non-linearity (*p* > 0.05) being interpreted as lack of support for significant deviation from linearity.

To check the robust associations between DII with all-cause and cardiovascular mortality of the participants with MASLD, three sets of sensitive analyses were conducted to validate the main findings. First, we excluded participants who died within 2 years after the interview, which could reduce the potential reverse causality between exposure and outcome. Second, we tested the association between DII with mortality outcomes of adults who were interviewed between 1999 and 2006 years, which could check the impact of the different cycles we have chosen on the association. Third, we also checked the mortality prediction value of DII in a more generalizable population by applying FLI ≥ 30 as the diagnostic criteria for SLD.

All statistical analyses of the present study were conducted by using the R software.^3^

## 3 Results

### 3.1 The baseline characteristics of adults with MASLD

Over the period from 1999 to 2018, the basic characteristics of the 8,833 participants enrolled in the study are summarized in Table 1. The median age of the study population was 52.00 years, and the median BMI was 32.98 kg/m2. Male participants accounted for 51.06% (4,510 cases) of the total population, while females made up 48.94% (4,323 cases). The majority of adults with MASLD were non-Hispanic White (3,888 cases, 44.02%), and non-Hispanic Black individuals constituted 20.23% of the population (1,787 cases). Over half of the participants (54.75%) had an educational level of high school or below, and 28.53% had a college education. Additionally, 61.49% of the participants were current smokers, and 7.85% were current drinkers. Regarding comorbidities, 21.83% of the participants had a history of DM, and 10.30% had hypertension. The median DII was 1.60. During the median follow-up period, there were 1,493 all-cause deaths and 473 cardiovascular-related deaths. Non-survivors were more likely to be male, older, non-Hispanic White, have lower educational levels, be unmarried, have comorbidities, tend to smoke, have a lower BMI, and lower socioeconomic status, and have a higher DII compared to survivors (all *p* < 0.05).

**Table 1:** The demographic characteristics of adult with MASLD in the present study.

Variables	Total (*n* = 8,833)	Survivors (*n* = 7,340, 83.10%)	Non-survivors (*n* = 1,493, 16.90%)	H/*χ*^2^	*p*
DII, M(Q_1_, Q_3_)	1.60 (0.06, 2.83)	1.56 (−0.01, 2.80)	1.74 (0.35, 2.96)	**14.10**	**<0.001**
Age, M(Q_1_, Q_3_)	52.00 (37.00, 65.00)	48.00 (34.00, 61.00)	70.00 (61.00, 77,00)	**1562.92**	**<0.001**
BMI, M(Q_1_, Q_3_)	32.98 (29.92, 37.20)	33.29 (30.20, 37.56)	31.51 (28.73, 35.42)	**137.02**	**<0.001**
WAIST, M(Q_1_, Q_3_)	109.90 (103.20, 118.80)	109.70 (103.10, 118.70)	110.50 (104.00, 119.00)	3.06	0.080
TC, M(Q_1_, Q_3_)	196.00 (170.00, 255.00)	197.00 (170.00, 225.00)	194.00 (167.00, 226.00)	1.64	0.200
TR, M(Q_1_, Q_3_)	145.00 (103.00, 207.00)	143.00 (101.00, 204.25)	154.00 (112.00, 222.00)	**38.62**	**<0.001**
ALT, M(Q_1_, Q_3_)	26.00 (18.00, 40.00)	26.00 (18.00, 29.00)	28.00 (19.00, 46.00)	**37.24**	**<0.001**
GLU, M(Q_1_, Q_3_)	104.00 (96.00, 118.40)	103.00 (95.00, 106.00)	111.00 (99.70, 135.70)	**206.19**	**<0.001**
FLI, M(Q_1_, Q_3_)	85.27 (73.73, 94.38)	85.36 (73.67, 94.52)	84.88 (74.05, 93.54)	0.75	0.388
HDL, M(Q_1_, Q_3_)	46.00 (39.00, 54.00)	46.00 (39.00, 54.00)	46.00 (39.00,54.00)	0.90	0.342
Gender, *n* (%)				**53.84**	**<0.001**
Male	4,510 (51.06)	3,618 (49.29)	892 (59.75)		
Female	4,323 (48.94)	3,722 (50.71)	601 (40.25)		
Race, *n* (%)				**175.66**	**<0.001**
Mexican American	1944 (22.00)	1,697 (23.12)	247 (16.54)		
Hispanic	760 (8.61)	692 (9.43)	68 (4.55)		
Non-Hispanic White	3,888 (44.02)	3,018 (41.12)	870 (58.27)		
Non-Hispanic Black	1787 (20.23)	1,511 (20.59)	276 (18.49)		
Others	454 (5.14)	422 (5.75)	32 (2.14)		
Education level, *n* (%)				**101.56**	**<0.001**
≤ High school	4,836 (54.75)	3,841 (52.33)	995 (66.64)		
College	2,520 (28.53)	2,200 (29.97)	320 (21.44)		
> College	1,477 (16.72)	1,299 (17.70)	178 (11.92)		
Marital status, *n* (%)				**11.96**	**<0.001**
Not married	3,291 (37.26)	2,675 (36.44)	616 (41.26)		
Married	5,542 (62.74)	4,665 (63.56)	877 (58.74)		
PIR, *n* (%)				**49.60**	**<0.001**
< 1.3	2,909 (32.93)	2,388 (32.53)	537 (35.96)		
1.3–3.5	3,470 (39.29)	2,814 (38.34)	650 (43.54)		
> 3.5	2,454 (27.78)	2,138 (29.13)	306 (20.50)		
Smoking, *n* (%)				**198.61**	**<0.001**
No	3,401 (38.51)	2,114 (28.80)	406 (27.20)		
Yes	5,432 (61.49)	5,226 (71.20)	1,087 (72.80)		
Alcohol use, *n* (%)				**13.37**	**<0.001**
No	8,140 (92.15)	6,729 (76.18)	1,411 (15.97)		
Yes	693 (7.85)	611 (15.97)	82 (0.93)		
Hypertension, *n* (%)				**92.87**	**<0.001**
No	7,923 (89.70)	6,687 (91.10)	1,236 (82.79)		
Yes	910 (10.30)	653 (8.90)	257 (17.21)		
DM, *n* (%)				**23.07**	**<0.001**
No	6,905 (78.17)	5,668 (77.22)	1,237 (82.85)		
Yes	1928 (21.83)	1,672 (22.78)	256 (17.15)		

M, median; Q, quartile; DII, Dietary Inflammatory Index; TC, total cholesterol; TR, triglyceride; HDL, high-density lipoprotein; GLU, glucose; ALT, glutamic-pyruvic transaminase; FLI, fatty liver index; BMI, body mass index; PIR, poverty income ratio; DM, diabetes mellitus. Non-normally distributed variables are displayed as median with 1^st^ and 3^rd^ quartile (M, Q1, and Q3). Categorical variables are displayed as numbers with weighted percentages (*n*, %). Bold values indicate statistical significance (*p* < 0.05).

### 3.2 Association between DII with mortality outcomes of adults with MASLD

**Figure 2:**
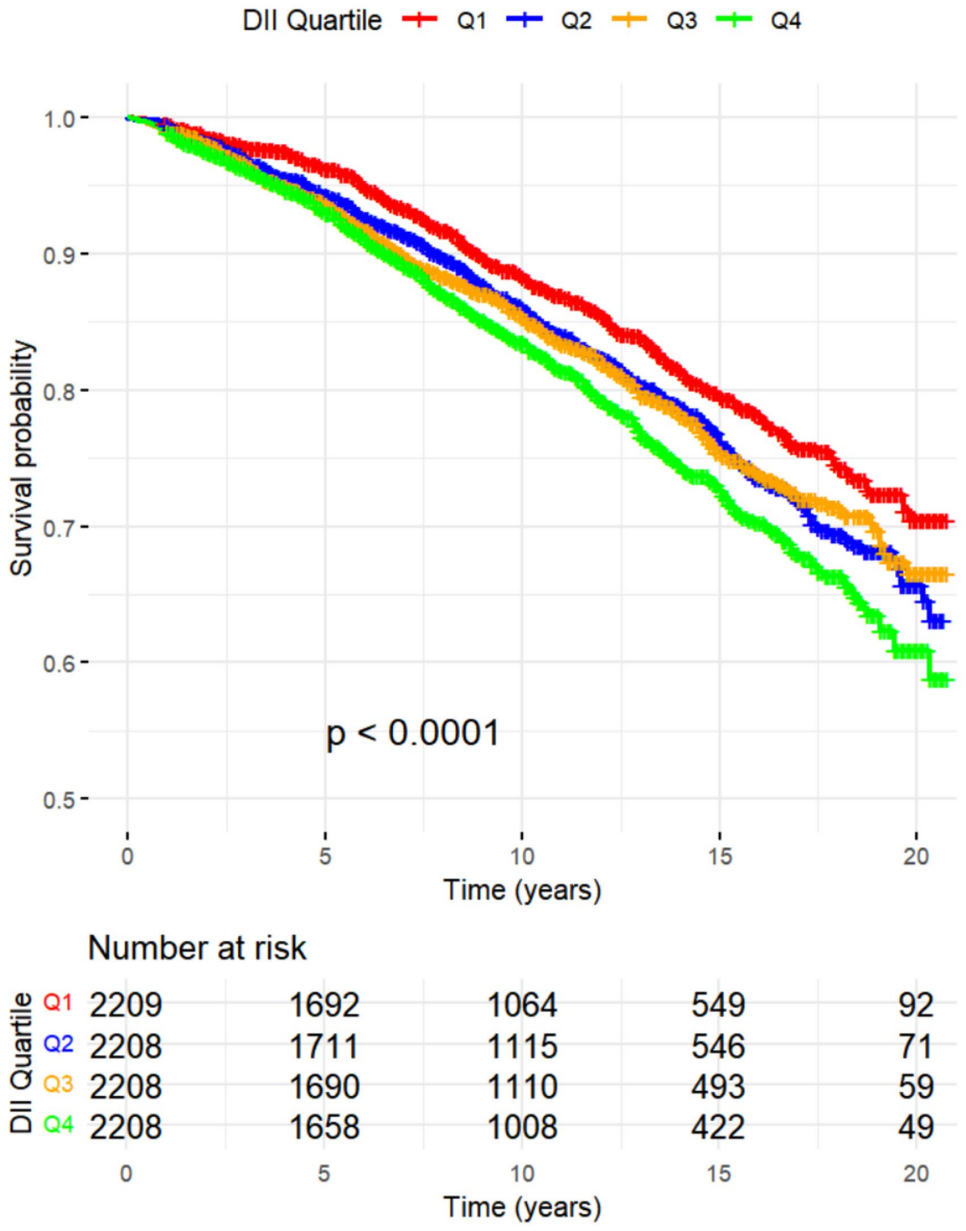
Kaplan–Meier Survival Curves for All-Cause Mortality by DII Quadrtiles with NHANES Sample Weight.

**Figure 3:**
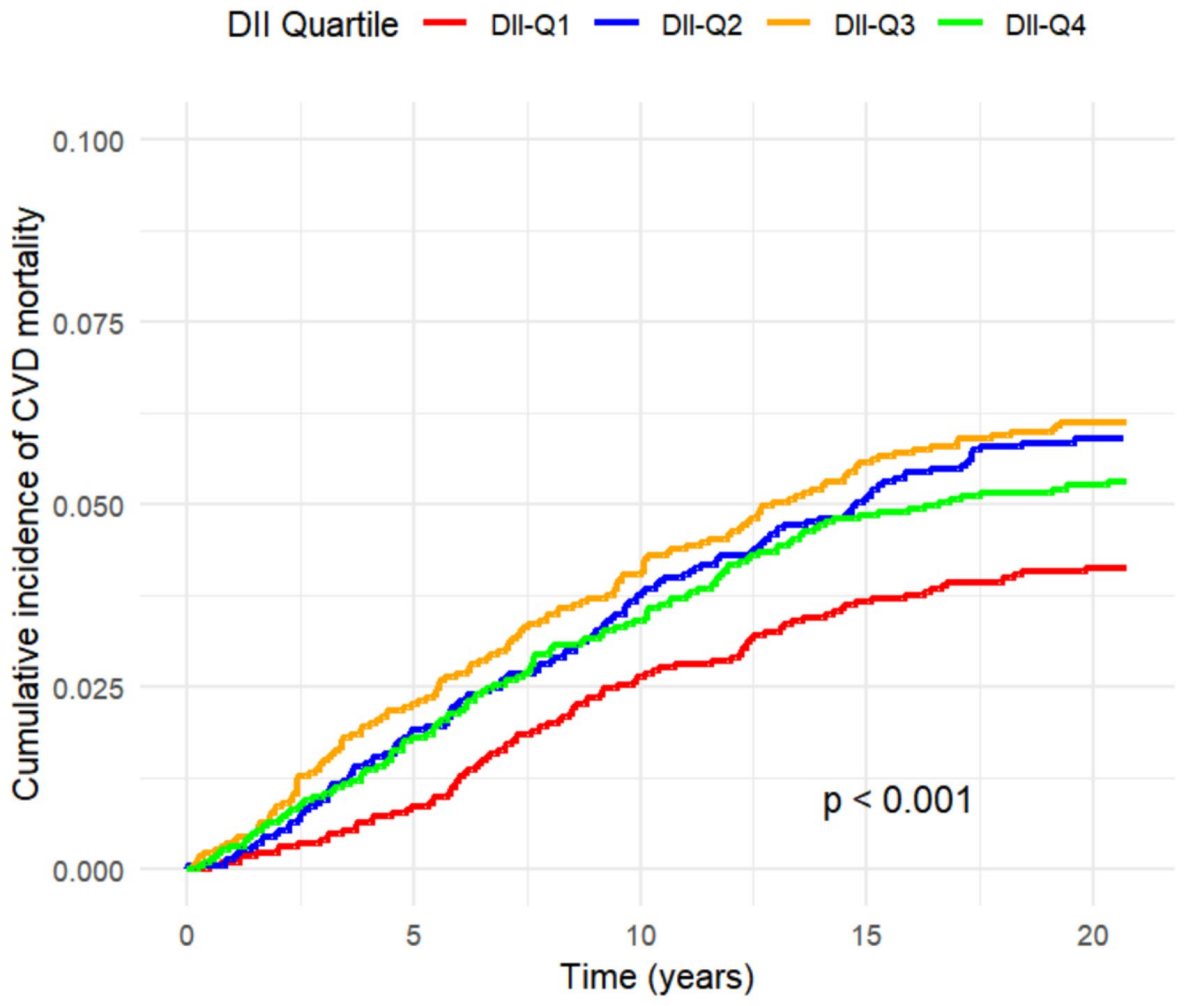
Cumulative Incidence Function for CVD Mortality by DII Quartiles Considering Competing Risks.

**Figure 4:**
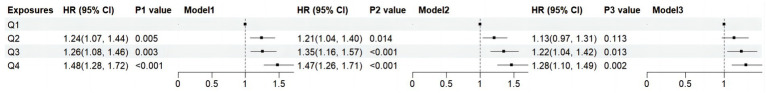
Forest plots show the association between DII with the all-cause mortality among adults with MASLD. DII, Dietary Inflammatory Index; HR: hazard ratio; CI: confidence interval; MASLD, metabolic dysfunction-associated steatotic liver disease; Q, quartile. Model 1: unadjusted; Model 2: adjusted for age, gender, race; Model 3: adjusted for age, gender, race, marital status, educational level, poverty income ratio, plasma glucose concentration, smoking status, alcohol use, hypertension, BMI, HDL, ALT, TR and TC.

**Figure 5:**
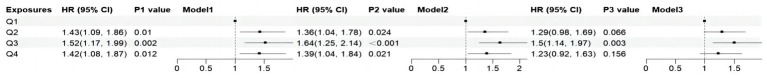
Forest plots show the association between DII with the cardiovascular mortality among adults with MASLD. DII, Dietary Inflammatory Index; HR, hazard ratio; CI, confidence interval; MASLD, metabolic dysfunction-associated steatotic liver disease; Q, quartile. Model 1: unadjusted; Model 2: adjusted for age, gender, race; Model 3: adjusted for age, gender, race, marital status, educational level, poverty income ratio, plasma glucose concentration, smoking status, alcohol use, hypertension, BMI, HDL, ALT, TR and TC.

**Figure 6:**
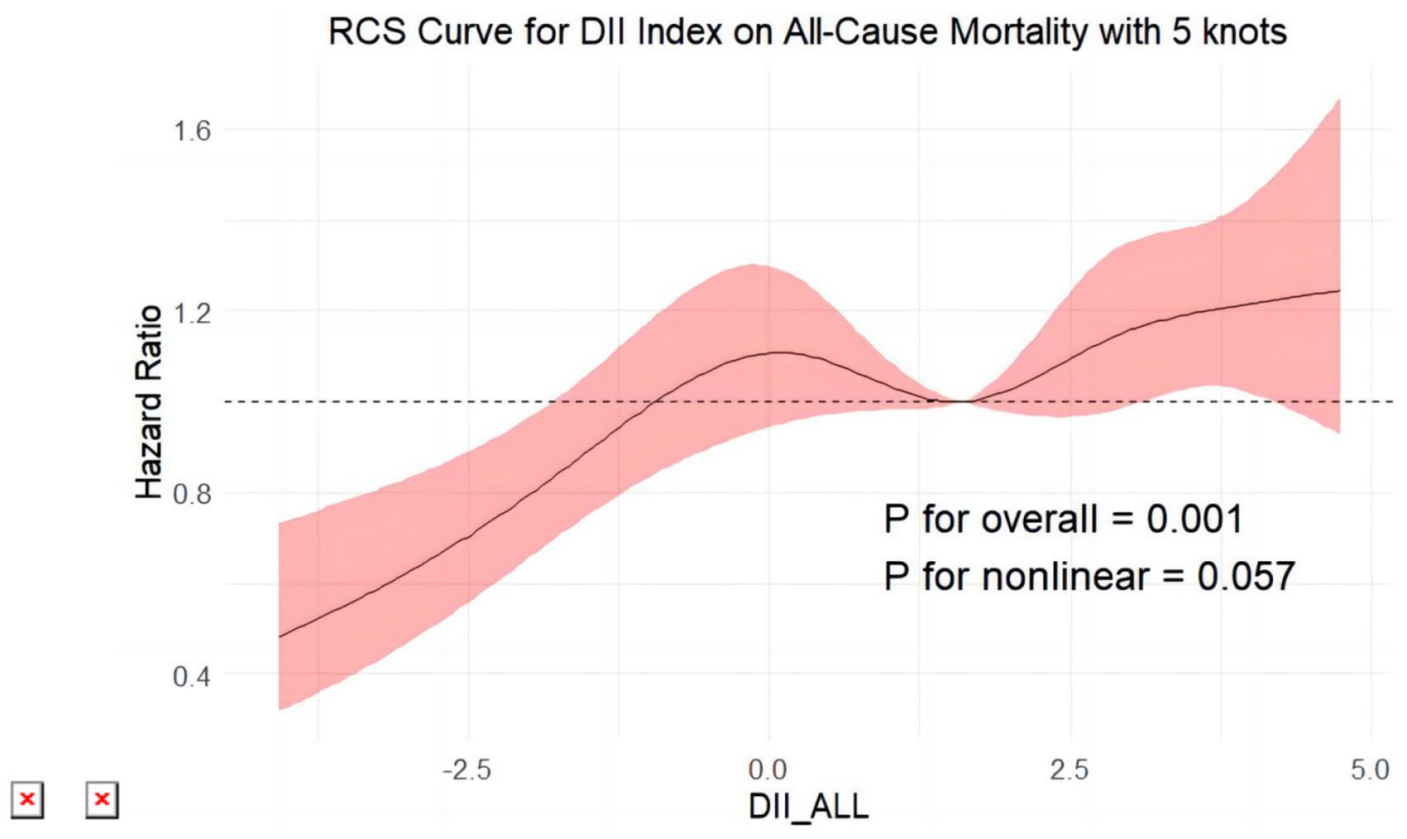
Restricted cubic splines reflect the dose-effect relationships between DII the all-cause mortality among adults with MASLD. DII, Dietary Inflammatory Index. The y-axis represents hazard ratio (HR), with the shaded area indicating the 95% confidence interval.

**Figure 7:**
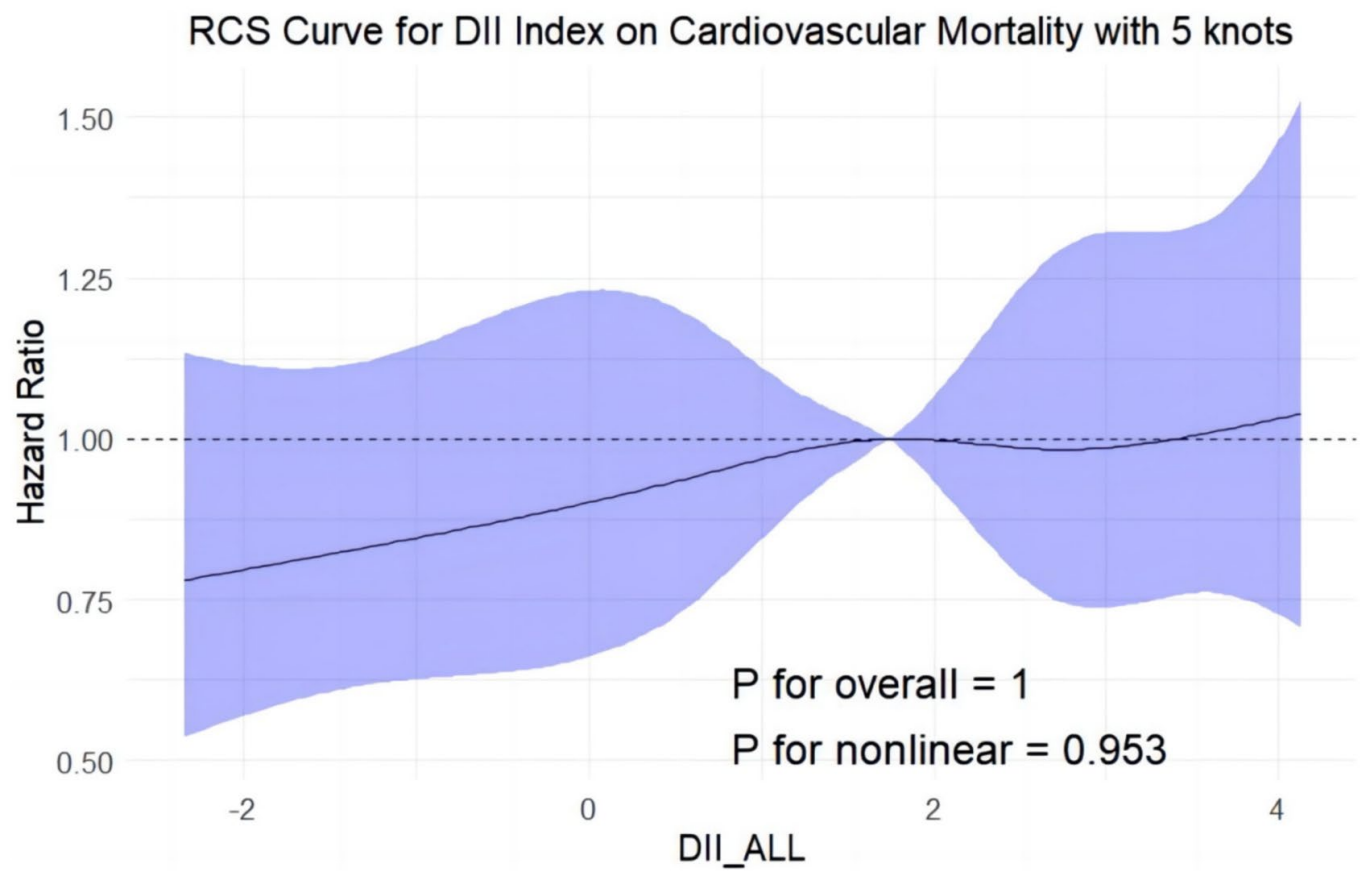
Restricted cubic splines reflect the dose-effect relationships between DII the cardiovascular mortality among adults with MASLD. DII, Dietary Inflammatory Index. The y-axis represents hazard ratio (HR), with the shaded area indicating the 95% confidence interval.

All-cause mortality was significantly associated with higher quartile levels of the DII among MASLD participants compared to those with lower quartile levels (*p* < 0.0001 by log-rank test, Figure 2). Consistently, participants with high levels of DII presented lower cardiovascular-specific survival probabilities (*p* = 0.012 by log-rank test) compared to other subgroups (Figure 3). The multivariate-adjusted Cox regression analysis revealed that the 4th quartile level of the DII was significantly associated with all-cause mortality [adjusted hazard ratio (aHR) = 1.28, 95% confidence interval (CI) = 1.10–1.49, *p* = 0.002]. However, the association between the high quartile levels of the DII and cardiovascular mortality was not statistically significant [aHR = 1.23, 95% CI = 0.92–1.63, *p* = 0.156] among adults with MASLD. When incorporated into a multivariate model including other clinically relevant variables, the DII yielded a C-index of 0.837 for all-cause mortality and 0.860 for cardiovascular mortality among participants with MASLD (Figures 4, 5). Finally, the multivariate-adjusted RCS analysis indicated a positive association between DII levels and mortality risk from all-cause and cardiovascular causes among MASLD adults. The *p*-values for non-linearity were 0.057 for all-cause mortality and 0.953 for cardiovascular mortality, suggesting insufficient evidence to support a non-linear relationship. (Figures 6, 7).

### 3.3 Sensitivity analyses

Subgroup analyses were conducted to determine whether demographic characteristics and clinical factors influenced the relationship between the Dietary Inflammatory Index (DII) and mortality outcomes in individuals with MASLD. The results showed consistent associations between DII and all-cause mortality across gender, age, BMI, marital status, and ALT levels (*P* for interaction >0.05). However, a significant interaction was observed with education level (*P* for interaction = 0.048), indicating that educational attainment may modify the association between DII and all-cause mortality (Supplementary Figure S1). For cardiovascular mortality, consistent results were observed across subgroups stratified by gender, race, marital status, BMI, smoking status, and ALT levels (*P* for interaction >0.05). A significant interaction effect was found for age (*P* for interaction = 0.036), suggesting a stronger association between DII and cardiovascular mortality in individuals under 65 years (Supplementary Figure S2).

Meanwhile, we conducted a series of sensitive analyses to check the robustness of the primary findings (Supplementary Tables S1–S3). First, consistent associations between the DII with all-causes as well as cardiovascular mortality were observed after excluding the participants who died within 2 years. Second, among adults interviewed in earlier cycles, the DII maintained significant associations with mortality outcomes in those with MASLD. Last, the DII demonstrated similar associations with mortality outcomes in adults subjected to more lenient inclusion criteria for the diagnosis of SLD (FLI ≥ 30).

## 4 Discussion

In the present investigation, we ascertained that a heightened DII is markedly linked to increased risks of all-cause mortality in adults diagnosed with MASLD. More precisely, MASLD subjects within the highest quartile of the DII were associated with an aHR of 1.28 for all-cause mortality (95% CI: 1.10–1.49, *p* = 0.002), denoting a substantial elevation in mortality risk compared to those with lower DII scores. Although a trend toward increased cardiovascular mortality was noted (aHR = 1.23, 95% CI: 0.92–1.63, *p* = 0.156), statistical significance was not achieved. When the DII was evaluated alongside additional clinical variables, the model demonstrated a C-index of 0.837 for all-cause mortality and 0.860 for cardiovascular mortality, underscoring the potential utility of integrating dietary and clinical factors in understanding mortality risk in MASLD populations. Further analysis using multivariate-adjusted RCS revealed a positive linear relationship between DII scores and mortality risks, suggesting a direct association. While the test for nonlinearity was not significant for cardiovascular mortality (*P* for nonlinearity = 0.953), it was marginally significant for all-cause mortality (*P* for nonlinearity = 0.057). These results emphasize the importance of considering dietary inflammation in the context of MASLD, paving the way for personalized nutritional interventions aimed at reducing disease burden and improving patient outcomes.

Prior research has demonstrated that a substantial association exists between higher DII levels and elevated inflammatory factors, particularly CRP, IL-6, and so on (26, 27). Therefore, the DII calculated in this study likely reflects the impact of the participants’ diets on their inflammatory status, making it a valuable metric for further analysis. Recent studies have highlighted that elevated DII scores are significantly correlated with a heightened risk of cardiovascular disease, diabetes, various common cancers, and an increase in all-cause mortality (16, 28). A study with 15,291 diabetic individuals in the U.S. found that after 45 months, those on a pro-inflammatory diet (DII > 0) had a 71% higher risk of all-cause mortality compared to those on an anti-inflammatory diet (DII < 0) (HR, 1.71; 95% CI, 1.13–2.58; *p* = 0.011) (29). A similar pattern was observed in hyperlipidemic patients, reinforcing the connection between dietary inflammation and increased mortality risk (30). A meta-analysis encompassing 15 studies across four continents revealed a consistent linear positive dose–response relationship between DII scores and all-cause mortality (31). The findings from the aforementioned studies on the DII in various populations mirror the results of this study. Another consideration is the impact of dietary fluctuations on metabolic health. Studies suggest that dietary inconsistency, especially alternating between inflammatory and anti-inflammatory diets, may exacerbate metabolic dysfunction, oxidative stress, and inflammation, potentially posing greater risks than a consistently high DII diet (32). Sudden dietary shifts have been linked to impaired insulin sensitivity and increased cytokine activity, both of which are central to MASLD pathogenesis (33, 34). Future research should investigate the long-term effects of dietary fluctuations.

There is still uncertainty as to why MASLD patients’ DII scores are associated with death risk. Potential mechanisms include the following: High DII diets exacerbate liver inflammation, speeding up the progression to fibrosis and cirrhosis by increasing systemic inflammation and insulin resistance common in MASLD. Additionally, such diets contribute to oxidative stress by increasing reactive oxygen species that damage hepatocytes (35, 36). The immune response is also implicated, pro-inflammatory diets activate the immune system, increasing cytokine production such as TNF-*α* and IL-6, which further aggravate liver damage and fibrosi. Moreover, the gut-liver axis plays a crucial role in MASLD progression, as dietary components shape gut microbiota composition. Pro-inflammatory diets (high in saturated fats, refined carbs, low fiber) disrupt microbiota balance, increasing intestinal permeability and lipopolysaccharide (LPS) translocation, which triggers hepatic inflammation via Kupffer cell activation (37). Conversely, fiber, polyphenols, and probiotics support gut homeostasis, increasing short-chain fatty acids (SCFAs) with anti-inflammatory and hepatoprotective effects. Anti-inflammatory and antioxidant-rich diets improve gut microbiota composition, reducing inflammation and oxidative stress (38). Targeting gut dysbiosis through dietary modifications may help reduce MASLD burden and improve outcomes. These interconnected mechanisms underscore the significant impact of dietary choices on the health outcomes of individuals with MASLD.

In the subgroup analysis, a stronger association was observed between DII and all-cause mortality among individuals with higher educational attainment. While higher education is generally linked to greater health literacy and healthier dietary habits, research suggests that factors such as social stress, work burden, and mental health challenges among highly educated individuals may contribute to chronic inflammation, amplifying the impact of a pro-inflammatory diet on mortality risk (39). This finding may reflect broader socioeconomic influences on dietary inflammation rather than being solely attributable to nutritional literacy. Similarly, the stronger association of DII scores with cardiovascular mortality in younger adults may be attributed to their higher consumption of processed, pro-inflammatory foods, which are rich in refined sugars, saturated fats, and additives. Studies have shown that such diets elevate inflammatory markers like C-reactive protein (CRP) and interleukin-6 (IL-6), contributing to vascular damage, arterial stiffness, and worsening liver inflammation and fibrosis, thereby accelerating MASLD progression (40). Prolonged exposure to these foods also induces oxidative stress, further damaging endothelial cells and promoting plaque formation. In contrast, older adults are more likely to adopt dietary adjustments as part of chronic disease management, including anti-inflammatory eating patterns like the Mediterranean or DASH diets, which reduce inflammation and oxidative stress (41).

The current study has several strengths. First, it is the first to investigate the prognostic implications of the DII on mortality outcomes among U.S. adults with MASLD. Second, the analysis is based on a robust dataset from NHANES, which provides a large, nationally representative sample and extensive follow-up data over two decades. Last, the study’s methodological rigor is evident through the comprehensive use of sensitivity analyses, which verify the stability and robustness of the results across various conditions and adjustments.

Some limitations need to be addressed in future studies. Potential selection bias in this study arises from the exclusion of participants with missing data on MASLD status and DII. However, the use of multiple imputation for covariates with minor missingness helps mitigate the impact of missing data. Additionally, Potential unmeasured confounders for this study include medication use and pre-existing CVD or other comorbidities. However, they may be correlated with observed confounders, which could reduce unmeasured confounding. Survival bias is also possible, as participants with higher DII who survived until the NHANES survey may represent a healthier subset, potentially underestimating the true association with mortality. Measurement errors are possible but likely minimal due to NHANES’ robust design and methods. Future studies incorporating clinically verified data, additional confounders, left truncation adjustments, or longitudinal follow-up from MASLD diagnosis could enhance the validity of these associations.

In conclusion, our findings suggest that elevated levels of DII are associated with increased risks of all-cause and cardiovascular mortality in U.S. adults with MASLD. Furthermore, DII demonstrates an enhanced associative effect on mortality outcomes in adults with MASLD, making it a simple and easily calculable clinical biomaker for managing MASLD.

## Data Availability

The datasets presented in this study can be found in online repositories. The names of the repository/repositories and accession number(s) can be found in the article/Supplementary material.

## Footnotes

^1^ https://www.cdc.gov/nchs/nhanes/

^2^ https://wwwn.cdc.gov/nchs/nhanes/tutorials/weighting.aspx

^3^version 4.2.3, https://www.r-project.org/
