# Supplementary material for: Association of dietary inflammatory index on all-cause and cardiovascular mortality in U.S. adults with metabolic dysfunction associated steatotic liver disease
Source: Front Nutr. 2025 Apr 1;12:1478165. doi: 10.3389/fnut.2025.1478165 (PMC12001832; doi:10.3389/fnut.2025.1478165)
Supplement: Supplementary file 1 [file Table_1.DOCX]

Supplementary Material

***Association of Dietary Inflammatory Index on All-Cause and Cardiovascular Mortality in U.S. Adults with Metabolic Dysfunction Associated Steatotic Liver Disease***


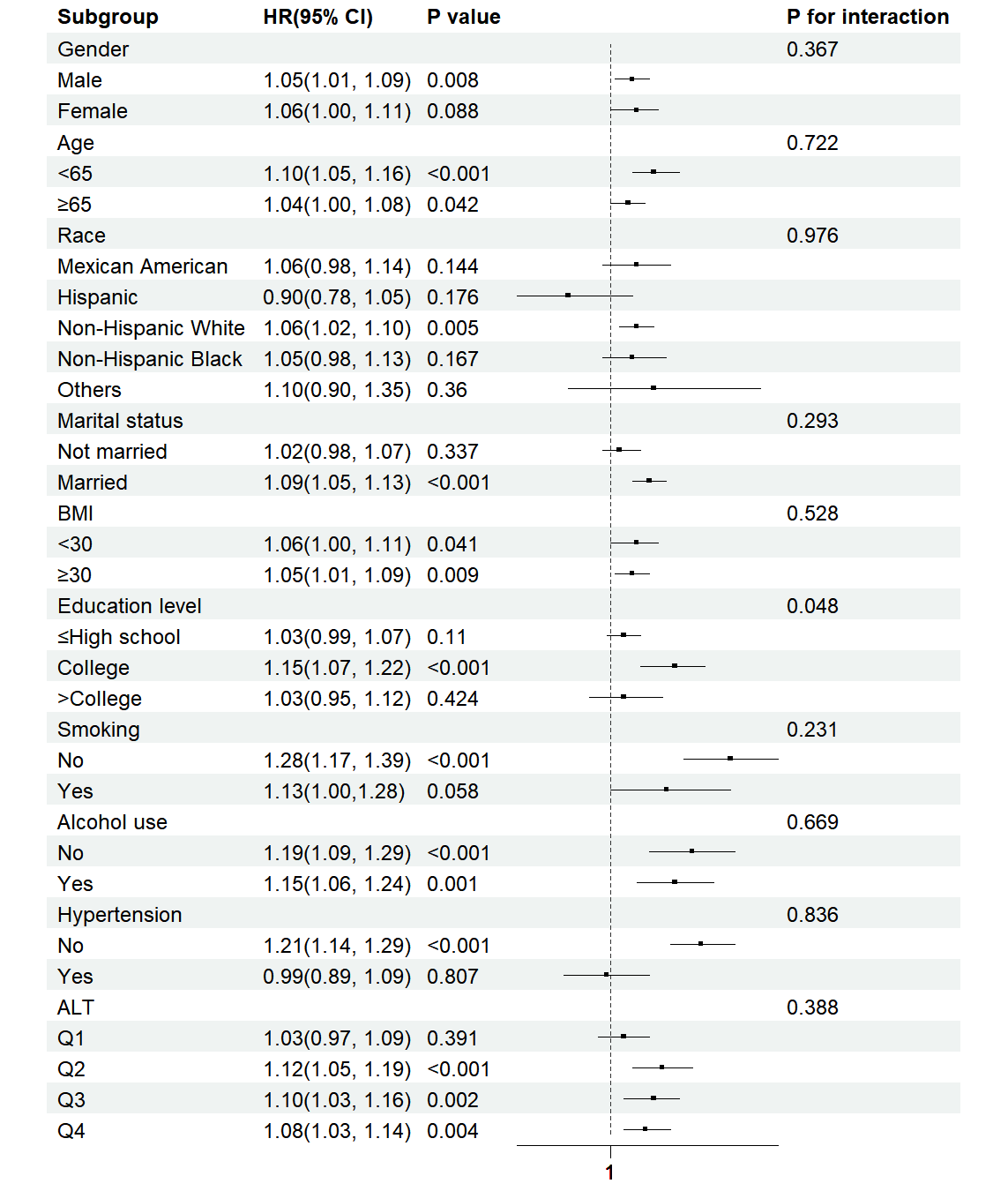


**Figure S1.** Association between DII index with all-cause mortality among adults with MASLD in different subgroups.


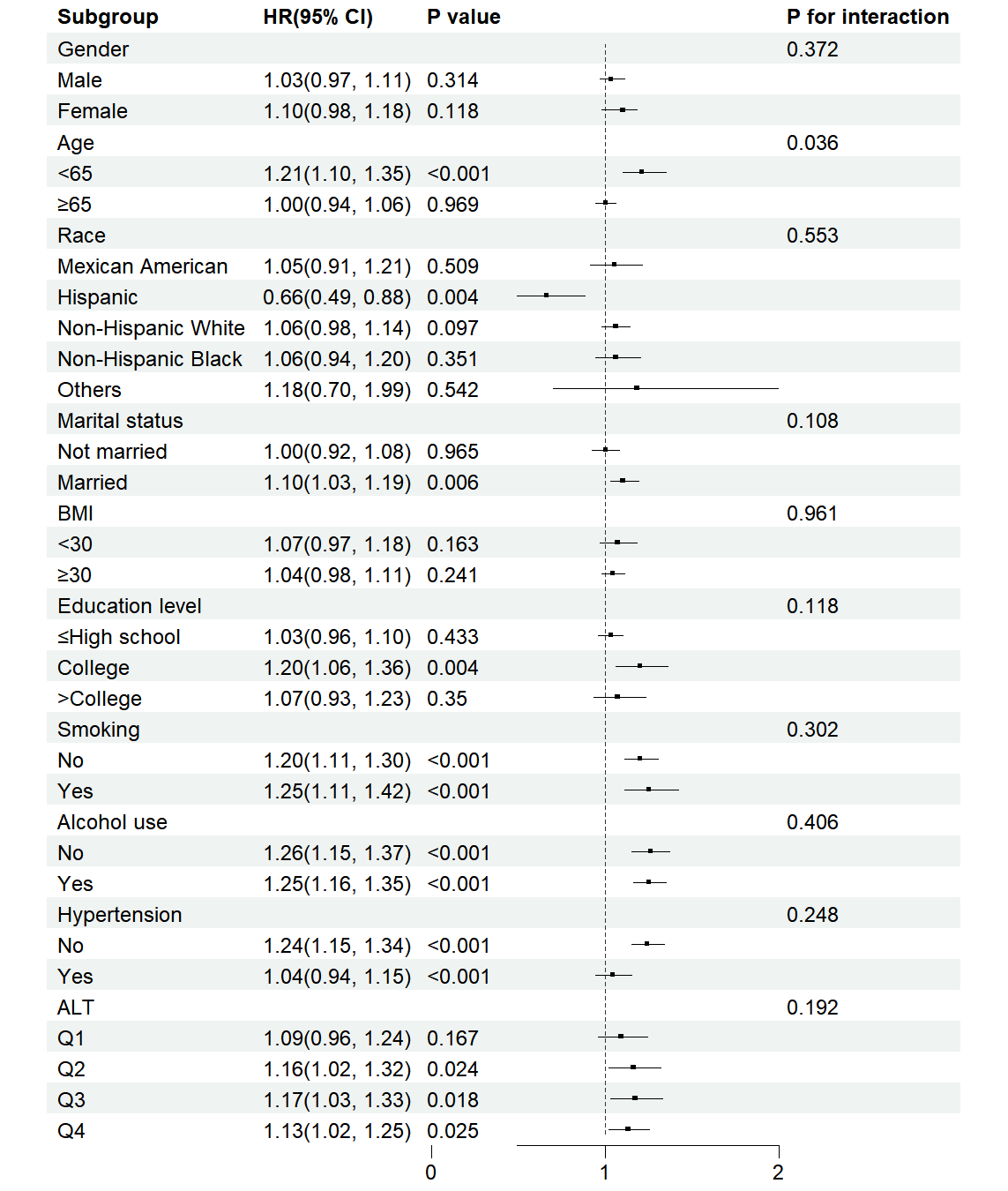


**Figure S2.** Association between DII index with cardiovasular mortality among adults with MASLD in different subgroups.

**Table S1.** Sensitive analysis to evaluate the association between the DII with the mortality outcomes of adults with MASLD by excluding participants who died within 2 years.

| **Subgroup** | **Model 1** | | **Model 2** | | **Model 3** | |
| --- | --- | --- | --- | --- | --- | --- |
|  | **HR (95% CI)** | ***P*** | **HR (95% CI)** | ***P*** | **HR (95% CI)** | ***P*** |
| **All-cause mortality** | | | | | | |
| Q1 | Reference | | Reference | | Reference | |
| Q2 | 1.23.(1.05-1.44) | **0.010** | 1.19(1.01-1.39) | **0.035** | 1.17(1.00-1.38) | **0.049** |
| Q3 | 1.24(1.06-1.46) | **0.008** | 1.34(1.14-1.57) | **<.001** | 1.19(1.02-1.40) | **0.032** |
| Q4 | 1.46(1.25-1.71) | **<.001** | 1.44(1.23-1.69) | **<.001** | 1.38(1.17-1.62) | **<.001** |
| **Cardiovascular mortality** | | | | | | |
| Q1 | Reference | | Reference | | Reference | |
| Q2 | 1.41(1.07-1.86) | **0.017** | 1.34(1.01-1.77) | **0.043** | 1.33(1.01-1.77) | **0.045** |
| Q3 | 1.42(1.08-1.88) | **0.014** | 1.54(1.16-2.05) | **0.003** | 1.37(1.03-1.82) | **0.030** |
| Q4 | 1.36(1.01-1.81) | **0.040** | 1.32(0.99-1.78) | 0.062 | 1.28(0.95-1.73) | 0.099 |

Abbreviation: DII: Dietary Inflammatory Index; HR: hazard ratio; CI: confidence interval; Q: quartile.

Model 1: unadjusted; Model 2: adjusted for age, gender, race; Model 3: adjusted for age, gender, race, marital status, educational level, poverty income ratio, plasma glucose concentration, alcohol use, BMI, HDL, ALT, TR and TC.

**Table S2.** Sensitive analysis to evaluate the association between DII with the mortality outcomes of adults with MASLD by only including participants from 1999 to 2006.

| **Subgroup** | **Model 1** | | **Model 2** | | **Model 3** | |
| --- | --- | --- | --- | --- | --- | --- |
|  | **HR (95% CI)** | ***P*** | **HR (95% CI)** | ***P*** | **HR (95% CI)** | ***P*** |
| **All-cause mortality** | | | | | | |
| Q1 | Reference | | Reference | | Reference | |
| Q2 | 1.31(1.09-1.57) | **0.003** | 1.21(1.01-1.46) | **0.041** | 1.26(1.06-1.52) | **0.015** |
| Q3 | 1.28(1.06-1.52) | **0.009** | 1.36(1.13-1.64) | **0.001** | 1.25(1.03-1.50) | **0.025** |
| Q4 | 1.53(1.28-1.84) | **<.001** | 1.54(1.27-1.86) | **<.001** | 1.46(1.21-1.76) | **<.001** |
| **Cardiovascular mortality** | | | | | | |
| Q1 | Reference | | Reference | | Reference | |
| Q2 | 1.63(1.27-2.27) | **0.004** | 1.48(1.06-2.07) | **0.022** | 1.52(1.09-2.14) | **0.014** |
| Q3 | 1.63(1.17-2.28) | **0.004** | 1.74(1.24-2.44) | **0.001** | 1.59(1.13-2.24) | **0.008** |
| Q4 | 1.70(1.22-2.37) | **0.002** | 1.71(1.22-2.40) | **0.002** | 1.63(1.15-2.30) | **0.006** |

Abbreviation: DII: Dietary Inflammatory Index; MASLD, metabolic dysfunction-associated steatotic liver disease; HR: hazard ratio; CI: confidence interval; Q: quartile.

Model 1: unadjusted; Model 2: adjusted for age, gender, race; Model 3: adjusted for age, gender, race, marital status, educational level, poverty income ratio, plasma glucose concentration, alcohol use, BMI, HDL, ALT, TR and TC.

**Table S3.** Sensitive analysis to evaluate the association between DII with the mortality outcomes of adults with FLI ≥ 30.

| **Subgroup** | **Model 1** | | **Model 2** | | **Model 3** | |
| --- | --- | --- | --- | --- | --- | --- |
|  | **HR (95% CI)** | ***P*** | **HR (95% CI)** | ***P*** | **HR (95% CI)** | ***P*** |
| **All-cause mortality** | | | | | | |
| Q1 | Reference | | Reference | | Reference | |
| Q2 | 1.24(1.07-1.44) | **0.004** | 1.21(1.04-1.40) | **0.014** | 1.19(1.03-1.39) | **0.022** |
| Q3 | 1.26(1.08-1.46) | **0.003** | 1.35(1.16-1.58) | **<.001** | 1.21(1.04-1.41) | **0.015** |
| Q4 | 1.48(1.28-1.72) | **<.001** | 1.47(1.26-1.71) | **<.001** | 1.40(1.20-1.63) | **<.001** |
| **Cardiovascular mortality** | | | | | | |
| Q1 | Reference | | Reference | | Reference | |
| Q2 | 1.43(1.09-1.86) | **0.010** | 1.36(1.04-1.78) | **0.024** | 1.37(1.04-1.79) | **0.023** |
| Q3 | 1.52(1.17-1.99) | **0.002** | 1.63(1.25-2.14) | **<.001** | 1.48(1.13-1.94) | **0.005** |
| Q4 | 1.42(1.08-1.87) | **0.012** | 1.39(1.05-1.84) | **0.021** | 1.36(1.02-1.81) | **0.033** |

Abbreviation: DII: Dietary Inflammatory Index; HR: hazard ratio; CI: confidence interval; Q: quartile.

Model 1: unadjusted; Model 2: adjusted for age, gender, race; Model 3: adjusted for age, gender, race, marital status, educational level, poverty income ratio, plasma glucose concentration, alcohol use, BMI, HDL, ALT, TR and TC
